# Enamel Chisels

**Published:** 1883-09

**Authors:** 


### ENAMEL CHISELS.

For a short time we have been using a set of Dr. E. Parmly Brown's Enamel Chisels, and now wonder how we have been all our life doing without them. There are six in the set, and each has double ends, making twelve chisels altogether. Their forms are quite unlike any others that we have met, and they are adapted to every position in which they might be needed. At first view they appeared too large and heavy, but experience has taught that this was a mistaken estimate. Their temper and workmanship are all that can be desired, and they are valuable assistants to any one who wishes to perform thorough operations.

